# The Physiological Action of Alcohol

**Published:** 1876-12

**Authors:** 


### EDITORIAL, ETC.

*The Physiological Action of Alcohol.*—At the late meeting of the American Dental Association, held in Philadelphia, the report of the special committee on " The Health of the Dentist," and of which Dr. John Allen was chairman, gave rise to considerable discusssion, especially concerning the use of narcotics and stimulants by the dental practitioner whose nervo-muscular system is so greatly taxed ; the value of alcohol as medicine was debated, some of the gentlemen advocating its use as beneficial, and regarding it as a main reliance in certain pulmonary affections, while others were not willing to admit those views. The following articles on the physiological and therapeutical action of this agent will prove interesting :

Dr. T. Brunton, in the *Practitioner*, says:

1. Alcohol in small quantities increases the secretion of the gastric juice and the movements of the stomach, and thus aids digestion. Although unnecessary in health, it is useful in exhaustion and debility.

2. It increases the force and frequency of the pulps, by acting reflexly through the nerves of the stomach.

3. In large doses it impairs digestion by over-irritating the stomach.

4. It may produce death reflexly by shock.

5. After absorption into the blood it lessens the oxidizing power of the red blood corpuscles. This property renders it useful in reducing temperature. When constantly, or even frequently, present in the blood, it causes accumulation of fat, and fatty degeneration of organs.

6. It undergoes cumbustion in the body, maintains or increases the body weight, and prolongs life on an unsufficient diet. It is therefore entitled to be reckoned as a food.

7. If large doses are taken, part of it is excreted unchanged.

8. It dilates the blood-vessels, increases the force and frequency of the heart by its action on the nervous centres, to which it is conveyed by the blood, imparts a feeling of comfort, and facilitates bodily and mental labor. It does not give additional strength, but merely enables a man to draw upon his reserve energy. It may thus give assistance in a single effort, but not prolonged exertions.

9. The same is the case with the heart; but in diseases alcohol frequently slows instead of quickening the pulsations of this organ, and thus economizes instead of expending its reserve energy.

10. By dilating the vessels of the skin, alcohol warms the surface at the expense of the internal organs. It is thus injurious when taken during exposure to cold, but beneficial when taken after the exposure is over, as it tends to prevent congestion of internal organs.

11. The symptoms of intoxication are due to paralysis of the nervous centres; the cerebrum and cerebellum being first affected, then the cord, and lastly the medulla oblongata. It is through paralysis of the medulla that alcohol usually causes death.

12. The apparent immunity which drunken men enjoy from the usual effects of serious accidents, is due to paralysis of the nervous mechanism, through which shock would be produced in a sober condition.

Dr. Norman Kerr, before the British Medical Association, protests against the too common use of alcohol in medical practice, and concluded by suggesting the three following rules of practice;—"first never to order alcohol, unless it was absolutely necessary; second, to order alcohol in a spirit of wine mixture if that would answer; and third, when fermented or spirituous liquor was required, to order it in precise doses, such as 'teaspoonfuls,' on the understanding that the medicine was not to be repeated unless the prescription was renewed."

